# Development of an intervention to expedite cancer diagnosis through primary care: a protocol

**DOI:** 10.3399/bjgpopen18X101595

**Published:** 2018-09-05

**Authors:** Marian Andrei Stanciu, Rebecca-Jane Law, Sadia Nafees, Maggie Hendry, Seow Tien Yeo, Julia Hiscock, Ruth Lewis, Rhiannon T Edwards, Nefyn H Williams, Katherine Brain, Paul Brocklehurst, Andrew Carson-Stevens, Sunil Dolwani, Jon Emery, William Hamilton, Zoe Hoare, Georgios Lyratzopoulos, Greg Rubin, Stephanie Smits, Peter Vedsted, Fiona Walter, Clare Wilkinson, Richard D Neal, The WICKED Team on behalf of

**Affiliations:** 1 Research Officer, North Wales Centre for Primary Care Research, Bangor University, Wrexham, UK; 2 Research Officer, North Wales Centre for Primary Care Research, Bangor University, Wrexham, UK; 3 Research Project Support Officer, North Wales Centre for Primary Care Research, Bangor University, Wrexham, UK; 4 Research Fellow, North Wales Centre for Primary Care Research, Bangor University, Wrexham, UK; 5 Research Fellow, Centre for Health Economics and Medicines Evaluation (CHEME), Bangor University, Bangor, UK; 6 Research Fellow, North Wales Centre for Primary Care Research, Bangor University, Wrexham, UK; 7 Research Fellow in Health Sciences Research, North Wales Centre for Primary Care Research, Bangor University, Wrexham, UK; 8 Professor of Health Economics, Centre for Health Economics and Medicines Evaluation (CHEME), Bangor University, Bangor, UK; 9 Professor in Primary Care, Department of Health Services Research, University of Liverpool, Liverpool, UK; 10 Professor, Division of Population Medicine, School of Medicine, Cardiff University, Cardiff, UK; 11 Professor in Health Services Research, North Wales Organisation for Randomised Trials in Health, Bangor University, Bangor, UK; 12 Clinical Reader, Division of Population Medicine, School of Medicine, Cardiff University, Cardiff, UK; 13 Senior Clinical Lecturer, Division of Population Medicine, School of Medicine, Cardiff University, Cardiff, UK; 14 Professor of Primary Care Cancer Research, Department of General Practice and Centre for Cancer Research, University of Melbourne, Melbourne, Australia; 15 Professor of Primary Care Diagnostics, Discovery Research Group, University of Exeter, Exeter, UK; 16 Principal Trial Statistician, North Wales Organisation for Randomised Trials in Health, Bangor University, Bangor, UK; 17 Professor of Cancer Epidemiology, Department of Behavioural Science and Health, University College London, London, UK; 18 Professor of General Practice and Primary Care, Institute of Health and Society, University of Newcastle, Newcastle, UK; 19 Research Associate, Division of Population Medicine, School of Medicine, Cardiff University, Cardiff, UK; 20 Professor, Research Director, Department of Public Health, Research Centre for Cancer Diagnosis, Research Unit for General Practice, Aarhus University, Aarhus, Denmark; 21 Professor, Research Director, Department of Clinical Medicine, University Clinic for Innovative Health Care Delivery, Silkeborg Hospital, Aarhus University, Aarhus, Denmark; 22 Principal Researcher in Primary Care Cancer Research, Primary Care Unit, Department of Public Health and Primary Care, University of Cambridge, Cambridge, UK; 23 Professor of General Practice, North Wales Centre for Primary Care Research, Bangor University, Wrexham, UK; 24 Professor of Primary Care Oncology, Academic Unit of Primary Care, Institute of Health Sciences, University of Leeds, Leeds, UK; 25 North Wales Centre for Primary Care Research, Bangor University, Wrexham, Wales

**Keywords:** primary care, general practitioners, early cancer diagnosis, hospital referral, realist synthesis, discrete choice experiment, survey, qualitative

## Abstract

**Background:**

GPs can play an important role in achieving earlier cancer diagnosis to improve patient outcomes, for example through prompt use of the urgent suspected cancer referral pathway. Barriers to early diagnosis include individual practitioner variation in knowledge, attitudes, beliefs, professional expectations, and norms.

**Aim:**

This programme of work (Wales Interventions and Cancer Knowledge about Early Diagnosis [WICKED]) will develop a behaviour change intervention to expedite diagnosis through primary care and contribute to improved cancer outcomes.

**Design & setting:**

Non-experimental mixed-method study with GPs and primary care practice teams from Wales.

**Method:**

Four work packages will inform the development of the behaviour change intervention. Work package 1 will identify relevant evidence-based interventions (systematic review of reviews) and will determine why interventions do or do not work, for whom, and in what circumstances (realist review). Work package 2 will assess cancer knowledge, attitudes, and behaviour of GPs, as well as primary care teams’ perspectives on cancer referral and investigation (GP survey, discrete choice experiment [DCE], interviews, and focus groups). Work package 3 will synthesise findings from earlier work packages using the behaviour change wheel as an overarching theoretical framework to guide intervention development. Work package 4 will test the feasibility and acceptability of the intervention, and determine methods for measuring costs and effects of subsequent behaviour change in a randomised feasibility trial.

**Results:**

The findings will inform the design of a future effectiveness trial, with concurrent economic evaluation, aimed at earlier diagnosis.

**Conclusion:**

This comprehensive, evidence-based programme will develop a complex GP behaviour change intervention to expedite the diagnosis of symptomatic cancer, and may be applicable to countries with similar healthcare systems.

## How this fits in

Cancer outcomes can be improved through earlier diagnosis and GPs can play a greater role, especially in expediting the diagnosis of symptomatic cancer. Clinician behaviour change is notoriously complex. This study will use an established framework (behaviour change wheel) to develop a complex GP behaviour change intervention to expedite the diagnosis of symptomatic cancer. There are a number of initiatives and interventions to improve cancer diagnosis, but none of these are targeted specifically at changing GPs’ behaviour.

## Introduction

Countries with well-developed national healthcare systems, such as the UK, can still have relatively poor cancer outcomes.^1–3^ Late diagnosis is a major contributor to poorer cancer outcomes;^4^ timely diagnosis leads to better outcomes.^5^ The role and importance of primary care in the early diagnosis of cancer is well established.^6–8^ The Aarhus statement defines the primary care interval as the period between the first presentation in general practice and the point of first referral to specialist care, during which primary care plays the major role in expediting cancer investigation and subsequent diagnosis.^9^

In healthcare systems without direct access to specialist medical services, patients with potential symptoms of cancer are first seen by their GPs and then referred for specialist care and investigations. Several healthcare systems operate a fast-track referral pathway for patients meeting predefined clinical criteria for suspected cancer.^10^ Although there is emerging evidence that implementing the urgent referral pathway has improved survival rates, these decisions still involve significant levels of clinical acumen, judgement, and risk assessment, and there is usually a large variation in use across practices, implying a quality gradient.^11–14^ Evidence from England shows that, across all cancers, an increasing number of patients are diagnosed through urgent referral routes (37% in 2015, and up to 52% in 2014 in some practices).^15^ However, in the National Cancer Diagnosis Audit, GPs reported diagnostic delays to have occurred in 22% of cases due to patient, clinician, and system factors,^16^ and a large number of patients continue to have very long times to diagnosis.^17^

Changing behaviour in GPs is challenging, but educating or facilitating GPs to improve appropriate suspected cancer referrals appears key.^18^ Didactic education or passive dissemination alone rarely changes GPs’ behaviours but interactive education, reminder systems, and multifaceted interventions are more promising.^18,19^ Practice-specific audit, feedback, and skills training have potential to reduce the primary care interval.^20^ Such behaviour-change interventions work best when grounded in the needs, preferences, and priorities of GPs, and when informed by a theoretical understanding of the barriers and enablers to timely, appropriate cancer referral.^21,22^ Determinants of clinical cancer diagnostic behaviour among GPs are largely unknown: a primary care vignette survey of eleven jurisdictions in the International Cancer Benchmarking Partnership found that readiness to investigate or refer was not associated with GP demographics, practice, or healthcare system variables;^23^ a study of UK GPs using interactive simulated consultations online found a strong association between first diagnostic impressions and subsequent diagnoses and decisions;^24^ preliminary consultations with a number of clinical colleagues locally suggested that individual variation in knowledge, attitudes, and beliefs including professional expectations and norms might be important factors.

Research is urgently needed to find ways to optimise the quality of GPs’ knowledge, attitudes, and behaviour, regarding the identification, investigation, and referral of patients with cancer signs and symptoms. This research programme (WICKED) will develop and assess the feasibility of a complex behaviour change intervention, aimed at reducing the primary care interval for people subsequently diagnosed with cancer. This is aimed at all cancers as patients present in primary with often undifferentiated symptoms; hence there is potential benefit for both ‘easier to diagnose’ and ‘harder to diagnose’ cancers. A series of interconnected work packages have been designed to achieve the objectives of:

- identifying evidence-based interventions that aim to change behaviour in relation to identification and referral of suspected cancer cases in primary care;
- determining how elements of the interventions that achieve behaviour change work, for whom, and in what circumstances;
- understanding GPs’ personal beliefs and behaviour regarding cancer investigations and referrals;
- exploring GPs’ preferences for the attributes (characteristics) surrounding earlier diagnosis of cancer in primary care;
- exploring primary care team systems and norms about identifying, investigating, and referring patients with cancer signs and symptoms;
- selecting intervention elements with most promise for altering individual GP and primary care team behaviours;
- developing a multi-faceted behaviour change intervention to shift GP thinking and practice culture (the ‘ThinkCancer’ intervention);
- conducting a feasibility trial of the intervention compared with usual care, including a process evaluation and a health economic evaluation; and
- planning and designing a definitive randomised controlled trial (RCT) of the effectiveness and cost-effectiveness of the intervention (the ‘ThinkCancer’ trial).

## Method

**Figure 1.**
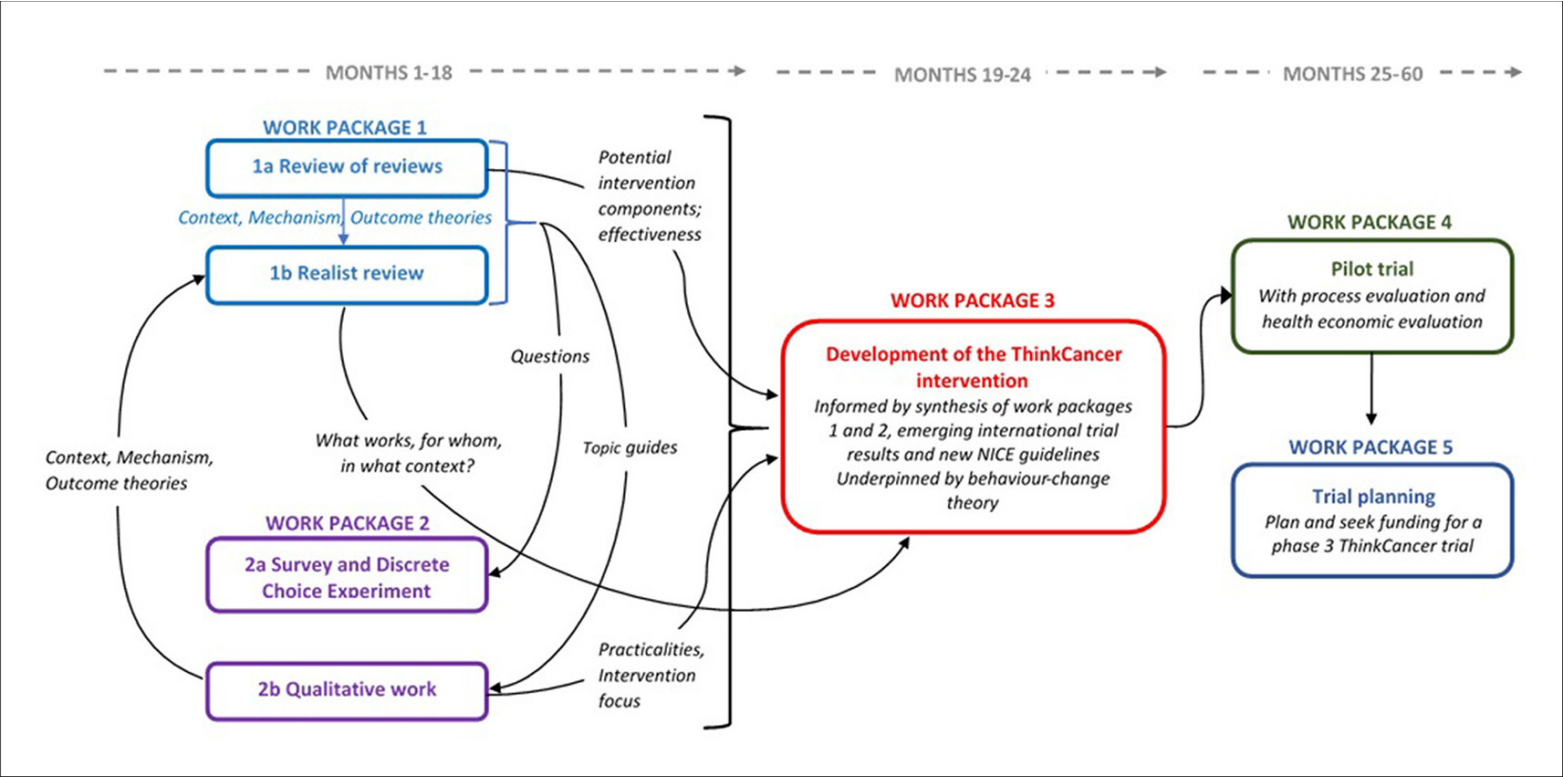
Diagram of the WICKED (Wales Interventions and Cancer Knowledge about Early Diagnosis) research programme

This research programme covers the first two phases of the Medical Research Council’s framework for the development of complex interventions:^25^ developing the intervention, and assessing feasibility. Four interconnected work packages comprise the programme (see Figure 1). A fifth work package will involve planning and seeking funding for a phase III definitive RCT.

### Work package 1a: review of reviews

The review of reviews will follow methodological guidelines for rigorous conduct and reporting.^26^ Its primary purpose will be to identify and describe the literature relating to the causes of longer primary care intervals, evidence-based interventions to address them, attributes that could be considered in the design of the final complex intervention, and barriers and facilitators associated with their implementation. It will provide source material for the development of initial programme theories — that is, hypotheses that explain how and why interventions work (or may not work), for whom, and in what contexts — which will then be tested in the realist review. Emerging findings from the review will also inform the development of questions for the survey and the identification of appropriate attributes for the DCE, and the development of the topic guides for the qualitative work.

MEDLINE, EMBASE, Cochrane Library, CINAHL, PsycINFO, and Web of Science will be searched. The search strategy will include thesaurus terms and synonyms for ‘primary care’, ‘cancer’, ‘interval’ plus ‘diagnosis or referral’, and ‘early’ plus ‘diagnosis or referral’, and a filter for systematic reviews (further information available from the authors on request).

The review will include any systematic reviews published in English, which examine the causes of longer primary care intervals, or describe interventions designed to reduce them at an individual practitioner or practice level. Interventions that target patients directly will not be included. Two reviewers will independently screen titles and abstracts to identify potentially relevant references, assess full-text papers for inclusion, and assess the quality of included reviews using the AMSTAR (A Measurement Tool to Assess Systematic Reviews) checklist for systematic reviews.^27^ Disagreements will be resolved by discussion. Taking a pragmatic approach appropriate to the purpose of the review (to inform the research programme as a whole, and in particular the realist review), and in keeping with rapid review methods, data extraction will be undertaken by one reviewer, and checked by a second.^28^ The findings will be analysed using structured tables and synthesised as a narrative synthesis.

### Work package 1b: realist review

The realist review will use a theory-driven approach to provide an explanatory account of what works in interventions designed to expedite cancer diagnosis in primary care, for whom, and in which circumstances.^29^ This will explain which factors might bring about behaviour or other change in relation to cancer investigation and referral. A list of barriers and facilitators to timely referral identified initially from background reading will be further developed and supported by evidence from the review of reviews, emerging findings of the qualitative work, and a scoping search of local initiatives. Based on this evidence matrix, and through engagement and discussion with stakeholders, an initial programme theory will be developed to explain what, how, and under which circumstances interventions might (or might not) work, expressed as a range of context, mechanism, and outcome configurations.^29^

To test and refine the programme theory, published literature and other relevant sources (such as reports and web pages) relating to each theory area will be searched. At least two reviewers will read relevant papers. Data will be extracted using a bespoke form and will include study details, intervention details, contextual details, and ‘fit’ with the programme theory. Relevant data collected from the qualitative interviews and focus groups (see work package 2 below) will also be used to explore GP and practice team perspectives.

Data synthesis will include all extracted data to test and refine the programme theories, and search for the relationships between and patterns of contexts, mechanisms, and outcomes. Findings will be reported in line with RAMESES publication standards.^30^ Both components of work package 1 are registered on the PROSPERO database.^31^

### Work package 2a: quantitative survey and DCE

The survey will measure the GPs’ cancer knowledge, investigation, and referral attitudes; their behaviours, learning needs, and preferences; and mechanisms supportive of change. These will be covered in the following sections:

- GP demographics; that is, sex, professional roles, and duration as a GP.
- Practice characteristics: patient list size, number of whole-time equivalent GPs, deprivation, teaching and training status.
- Areas of past clinical behaviour change and potential drivers of change.
- Capability, opportunity, and motivation regarding early cancer referral behaviour, as part of the Capability, Opportunity, Motivation–Behaviour (COM-B) model of the behaviour change wheel framework.^32^
- Knowledge of cancer symptoms and confidence in diagnosing cancer.
- Perceived role of GPs in diagnosing cancer, potential for GPs to improve cancer diagnosis.

A DCE, requiring individuals to state their preferences regarding hypothetical scenarios described by several attributes,^33^ will be employed to explore GPs’ preferences for different attributes (characteristics) surrounding earlier diagnosis of cancer in primary care. Preliminary work identified a broad number of attributes that could be considered in the design of a final complex intervention to improve earlier diagnosis of cancer in primary care. Both the survey and DCE will be piloted externally by a small sample of GPs (*N* = 10) from England (not included as participants in the study) and will be delivered together using the online SurveyMonkey platform (www.surveymonkey.com).

The survey data will first be analysed descriptively. Secondly, the relationship between areas of past clinical behaviour change, and potential behaviour drivers will be analysed using structural equation modelling (on reaching the target recruitment). Free-text data will be analysed using conventional content analysis.^34,35^ The DCE will determine the relative preferences of GPs for the individual attributes of interventions and the relative importance that they place on these different attributes.

All GPs in Wales (approximately 2000) will be invited to complete the survey and DCE, and the authors plan to recruit 200 participants. The 10% target recruitment rate is in line with the literature.^36^ Invitations to complete the survey and DCE will be sent by email, followed by two reminders, sent a week apart. Participating GPs will be offered a certificate of completion and a £30 gift voucher, with the option of returning £30 to charity.

### Work package 2b: qualitative interviews and focus groups

The qualitative component of the programme will explore GPs’ personal beliefs and behaviour, and practice team systems and norms, about identifying, investigating, and referring patients with cancer signs and symptoms. The overall approach will be aligned to the COM-B model and theoretical domains framework (TDF) components of the behaviour change wheel,^32^ and informed by integration of the findings from the literature reviews (work package 1).

In order to gain an in-depth account of individual experiences, beliefs, and behaviours, 20 GPs will be interviewed individually. In addition, to benefit from interactive discourse among members of the practice teams (including nursing, other healthcare, reception, and administrative staff) and to explore the shared experiences of team systems and culture, four focus groups with established practice teams will be conducted. Moderation techniques will facilitate contributions from all team members despite any pre-existing team hierarchies and dynamics.^37^ Both the interviews and focus groups will be conducted using a semi-structured topic guide, with flexibility to allow new areas to emerge and be discussed over the course of the qualitative enquiry. The GP interviews will last 30–﻿45 minutes and will be conducted over the telephone; focus group discussions will be practice-based and will last approximately 1 hour.

A purposive sampling method will be implemented for both interviews and focus groups to ensure a range in the sample of both GPs and practices. The sampling characteristics for the GP interviews will be: practice rurality (obtained using the rural–﻿urban classification for small area geographies),^38^ catchment area deprivation (Welsh index of multiple deprivation),^39^ and years since first medical qualification (obtained from the General Medical Council database).^40^ Sex characteristic distribution will be monitored to ensure representation from both male and female GPs. The sampling characteristics for the practice-level focus groups will be training practice status and practice rurality. Each focus group will involve 6–8 participants and include a range of GPs, practice nurses, practice managers, receptionists, and other team members.

Selected GPs and practices (via practice managers) will be invited to participate by email, with two reminders and a follow-up phone call. Based on recruitment rates and methods used in similar research,^41^ a response rate of approximately 10% is anticipated. GPs participating in the telephone interviews will receive a £30 gift voucher with the option of returning £30 to charity. Practices participating in a focus group will receive £250 for their involvement. All participants will receive a certificate of completion.

All interviews and focus groups will be audio recorded and transcribed verbatim. Analysis will use the framework approach and will follow the five stages of framework: familiarisation; thematic framework identification; indexing; charting; and mapping and interpretation.^42,43^ Interpretative analysis will be conducted by two experienced qualitative researchers, with additional participation of the wider research team in data interpretation workshops to discuss, develop, and refine the emerging themes. Later qualitative interviews may be used to test programme theories in the realist review.

### Work package 3: development of the intervention

**Figure 2.**
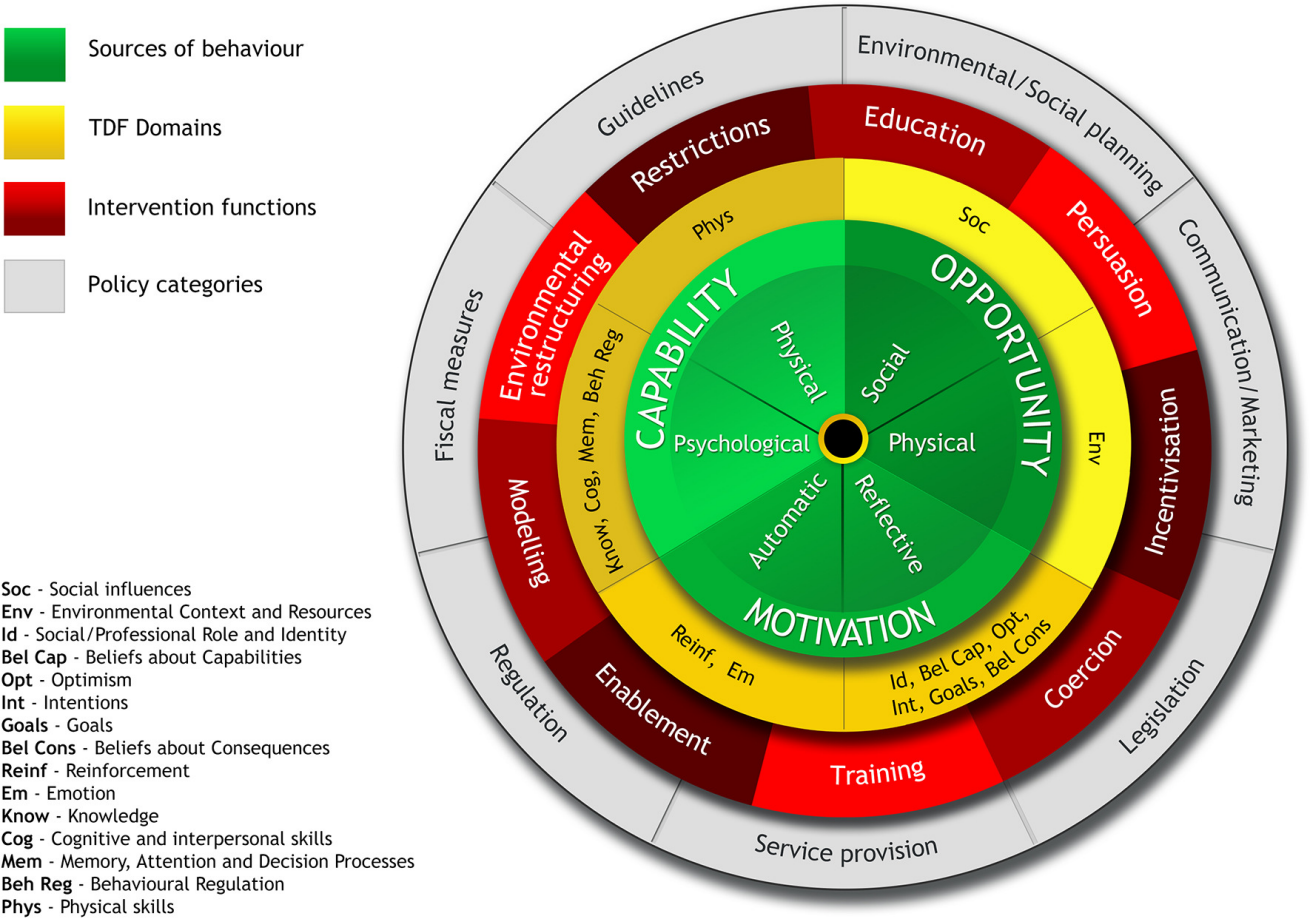
The behaviour change wheel, a framework for understanding behaviour (reproduced with permission from the authors)^44^

The behaviour change wheel comprises a suite of theory-based tools that are used to systematically guide the process of designing a complex behaviour change intervention, from initial identification of the target behaviour and factors that influence the behaviour, through to selecting the type, content, and mode of delivering an intervention, or set of interventions, that are most likely to produce change. Figure 2 illustrates the different layers that form the behaviour change wheel, with the COM-B model at its centre.^44^ COM-B describes how changing behaviour at the individual and/or system level is a result of changing one or more components of psychological and physical Capability, social and physical Opportunity, and automatic and reflective Motivation. The COM-B Model is encircled and supported by the TDF layer (represented in yellow), which provides a deeper level of understanding of the factors (or sources) that influence behaviour, and consists of fifteen theory domains that dovetail with the three COM-B components.^32^ Together, the COM-B model and TDF are used to identify what needs to change in order for the target behaviour to be achieved. The next layer (in red) describes nine potential functions that the intervention/s could serve, with each COM-B component and TDF domain linked to an intervention function. The outermost layer of the behaviour change wheel (shown in grey) describes seven policy categories, or actions taken by responsible authorities, that could enable or support the intervention. At each layer of the behaviour change wheel, complementary tools (for example, intervention functions matrix, APEASE criteria, and behaviour change techniques taxonomy) are available to guide developers’ decisions about intervention functions, specific content, and delivery.^44^

In work package 3, the behaviour change wheel will be used flexibly to guide the development of an intervention (or set of interventions) designed to change GPs’ behaviour regarding the way they think of, and act on, clinical presentations that could be cancer. The matrix of evidence (see work package 1, realist review) will be further refined, and relevant primary data from the quantitative survey and qualitative elements of work package 2 will be added iteratively, in order to document the factors that influence the target behaviour. The list of behavioural sources extracted from each source of evidence (work package 1 and work package 2) will then be mapped onto the COM-B components and TDF domains to identify which intervention functions are likely to be most effective in changing GPs’ behaviour. Once intervention functions are identified, the APEASE criteria will be used to support strategic judgements about the most relevant candidate intervention functions, and will involve considering the Affordability, Practicability, Effectiveness/cost-effectiveness, Acceptability, Side effects/safety and Equity of each intervention function in relation to GP behaviour. At this stage, the quantitative survey and DCE findings (work package 2a) will contribute to informing decisions about candidate intervention functions, by providing evidence about intervention users’ values and preferences regarding earlier diagnosis of cancer in primary care. The behaviour change techniques taxonomy is a further linked tool that can be used to support decisions about specific intervention content.^45^ The overall process of applying the behaviour change wheel will involve a core working group, with ‘data owners’ allocated to each source of work package 1 and work package 2 evidence. Where possible, tasks such as mapping the sources of behaviour to COM-B and TDF will initially be completed separately by individuals in order to reduce subjectivity, followed by core group discussion to arrive at a consensus. As the intervention develops, review will be undertaken by the programme advisory group, comprising patient representatives and clinical and academic partners.

### Work package 4: ThinkCancer feasibility trial

The final design of this phase will be determined by the precise nature of the intervention, the level at which it is likely to be applied, and consideration of the most likely primary outcome measure. It will be a phase II external pilot RCT. This will test the feasibility and acceptability of the intervention, including recruitment and retention rates, acceptability of elements of the intervention, the sample size calculation for a future full trial, and testing of economic evaluation methods. A concurrent process evaluation will also be conducted. Results will inform the design of a potential future phase III randomised controlled trial. The phase II pilot RCT will be run in close collaboration with the North Wales Organisation for Randomised Trials in Health.

## Discussion

### Summary

The findings of this research programme will be published in a peer-reviewed journal and will inform the definitive phase RCT of the intervention.^25^

### Strengths and limitations

Developing and implementing a behaviour change intervention in a diverse and changing health service is challenging and therefore requires a multi-dimensional approach to intervention development. The strength of this study is that it will use an integrated package of rigorously applied research methods to develop a complex primary care intervention which is evidence-based, takes into account local context, needs, and preferences, and is underpinned by behaviour change theory.

This innovative combination of methods draws from both realist and behaviour change perspectives. The realist element will develop and refine programme theory explaining why an intervention may or may not work, for whom, and in which circumstances. Behaviour change theory will be applied through use of the behaviour change wheel framework to consolidate the evidence and facilitate intervention development. This combined approach includes the use of multiple evidence sources and methodologies to develop the intervention. The realist element enables inductive theory building, and the systematic application of the behaviour change wheel will focus the theoretical perspective by specifying the likely mechanisms of change. Both methods are iterative in nature, and therefore a degree of flexibility in the conduct of this programme of work is desirable. The challenges will be to manage the elements of the research in a timely manner, so that the findings of individual work packages inform one another in a meaningful way, and to successfully integrate the methodological perspectives. Ongoing reflections will offer useful insight for future research.

### Comparison with existing literature

The authors believe that this programme of work is the first of its kind in this topic area and will therefore make an original research contribution.

### Implications for practice

This programme of work has potential impact and benefit for patients, GPs, and wider society in that the intervention(s) have the capacity to change GPs’ behaviour relating to how they investigate and refer people with potential symptoms of cancer. For patients, the authors would expect more timely investigation, referral, and diagnosis of symptomatic cancer. For primary care teams, improved quality of clinical care and more precisely targeted referrals and investigations for patients would be expected. A broader impact on the wider population would be expected through earlier stage diagnosis, subsequent improved cancer survival, and cost-effectiveness of the cancer diagnostic process.
